# Identifying gastric intestinal metaplasia risk based on clinical indicators: a machine learning predictive model based on the SHAP methodology

**DOI:** 10.3389/fphar.2025.1602191

**Published:** 2025-11-07

**Authors:** Yufen Wang, Jian Bi, Shunzhe Song, Ying Sun, Aixia Gong

**Affiliations:** 1 Department of Digestive Endoscopy, First Affiliated Hospital of Dalian Medical University, Dalian, China; 2 Department of Gastroenterology, First Affiliated Hospital of Dalian Medical University, Dalian, China

**Keywords:** gastric intestinal metaplasia, machine learning, clinical indicators, serological test, screening

## Abstract

**Background:**

Screening for gastric intestinal metaplasia (GIM) holds significant importance for the early detection of gastric cancer. To help clinicians identify high-risk GIM patients and determine the timing of gastric mucosal biopsy, we aim to develop a predictive model for the occurrence of GIM in patients.

**Methods:**

Patients were collected from the First Affiliated Hospital of Dalian Medical University, following rigorous inclusion and exclusion criteria. Initially, the VarSelRF algorithm identified independent variables linked to GIM development. We employed eight machine learning algorithms, including Decision Trees (DT), Elastic Net (ENet), K-Nearest Neighbors (KNN), LightGBM, Random Forest (RF), eXtreme Gradient Boosting (XGBoost), Support Vector Machine (SVM), and Multi-Layer Perceptron (MLP) to construct predictive models. Their performances were benchmarked using ROC curves, calibration curves, and decision curve analysis (DCA) curves. We also applied SHAP values to interpret the RF model, quantifying the contribution of each feature to predictions. Additionally, a web-based calculator was developed based on the RF model to facilitate practical clinical applications.

**Results:**

Among the 975 patients examined, 322 individuals were pathologically confirmed to have GIM. Eleven independent variables significantly contributed to GIM occurrence, including gastric mucosal atrophy, *H. pylori* infection, direct bilirubin (DBIL), creatinine (Crea), smoking and alcohol history, gender, alanine aminotransferase (ALT), age, albumin/globulin ratio (ALB/GLO), and gamma-glutamyltransferase (GGT). The RF model demonstrated strong performance among the eight machine learning algorithms tested, achieving an AUC of 0.8167 in the testing dataset, along with a specificity of 85.5% and a sensitivity of 57.0%. The model’s interpretive capabilities were enhanced by SHAP values, which helped clinicians understand the decision-making process. The resulting web-based calculator serves as a practical tool for clinicians.

**Conclusion:**

This study highlights the innovative use of serological biomarkers to assess the risk of GIM. We found that certain markers related to liver and kidney function are strong predictors of GIM development. Additionally, the application of SHAP values improves the understanding of how features contribute to predictions, while the newly developed web-based calculator offers a practical tool for clinicians to evaluate GIM risk more easily.

## 1 Introduction

Gastric cancer is the sixth most common malignancy worldwide and the third leading cause of cancer-related deaths, imposing a significant economic burden globally (Bray et al., 2018). Patients with advanced gastric cancer commonly experience symptoms such as stomach pain, weight loss, anemia, and cachexia, which severely reduce their quality of life (Smyth et al., 2020). Despite surgery combined with postoperative adjuvant chemotherapy, the 5-year survival rate for patients with advanced gastric cancer remains below 30%. In contrast, early gastric cancer patients who receive timely treatment, such as endoscopic submucosal dissection (ESD), can achieve a 5-year survival rate as high as 90%–95% (Maruyama et al., 2006). However, the onset of early gastric cancer is usually subtle and easy to ignore. In that way, early identification of precancerous lesions is particularly important.

Gastric adenocarcinoma develops through a cascade that begins with chronic superficial gastritis, progresses to chronic atrophic gastritis, and then to intestinal metaplasia and dysplasia before culminating in adenocarcinoma. Regular monitoring of precancerous conditions, such as chronic atrophic gastritis and gastric intestinal metaplasia (GIM), is crucial for the timely detection of early gastric cancer. Intestinal metaplasia refers to the replacement of gastric mucosa with intestinal epithelial cells, leading to fundamental tissue changes (Leun et al., 2002). This process is pivotal in the transition from precancerous disease to malignancy (Song et al., 2015).

Currently, gastroscopy combined with tissue biopsy is the only golden standard for diagnosing GIM. However, due to its high cost, invasive nature, and high dependence on pathologists, patient compliance is low (Malfertheiner et al., 2017). Although auxiliary examinations such as imaging and biomarkers have relatively better compliance, their clinical diagnostic specificity is inconclusive. Therefore, there is an urgent need for an effective and easily accessible tool to predict intestinal metaplasia of the gastric mucosa at an early stage, helping clinicians decide when to perform gastric mucosal tissue biopsy.

Intestinal metaplasia results from the gradual replacement of gastric mucosal cells by intestinal epithelial cells, often linked to gastric mucosal gland atrophy and *H. pylori* infection (Li et al., 2018). Recent studies have demonstrated that *H. pylori* infection extends beyond localized gastric pathology and may affect distant organ function through systemic inflammatory pathways (Santos et al., 2020). The key virulence factor γ-glutamyltranspeptidase (GGT) of *H. pylori* catalyzes glutathione degradation in the gastric mucosa, generating reactive oxygen species (ROS) and activating pro-inflammatory pathways such as NF-κB (Chen et al., 2023). These inflammatory mediators enter the systemic circulation and can trigger systemic inflammatory responses, subsequently affecting the metabolic functions of organs including the liver and kidneys (Wang et al., 2014; Koenig and Seneff, 2015). Furthermore, reduced gastric acid secretion from mucosal atrophy elevates intragastric pH, promoting abnormal colonization of intestinal flora and increasing the risk of bile reflux, both of which contribute to the development and progression of intestinal metaplasia. Additionally, bile reflux can impair gastric mucosal repair mechanisms (Shi et al., 2022). Research by Shahid et al. has identified distinct serum protein profiles in patients with gastric cancer, gastric ulcers, and gastritis (Aziz et al., 2022). Studies have shown that kidney function markers (such as serum creatinine and blood urea nitrogen) in *H. pylori*-infected patients may undergo subtle changes that correlate with the degree of gastric mucosal atrophy. Therefore, serum hepatorenal function markers may serve as biomarkers reflecting systemic inflammation and oxidative stress, indirectly predicting the degree of gastric mucosal pathology to some extent. Hepatorenal function tests are routine clinical examinations with standardized detection methods, stable and reliable results, easily accessible data, and low cost. Compared to expensive endoscopic examinations, serological markers offer non-invasive and convenient advantages, making them more suitable for large-scale screening and early prediction. However, due to the complexity and diversity of these serological indicators, the sensitivity and specificity of a single indicator are limited. Therefore, it is necessary to comprehensively consider multiple factors and explore their predictive utility in GIM in depth.

Therefore, this study aims to develop a model for the early prediction of GIM by using common serum markers related to liver and kidney function, as well as potential risk factors for GIM. Candidate indicators include patients’ basic information, potential factors of known gastric-related diseases, serum markers of liver function and kidney function. In the modeling process, eight different machine learning algorithms were employed to construct the models, including Decision Tree (DT), Elastic Net (ENet), K-Nearest Neighbors (KNN), LightGBM, Random Forest (RF), eXtreme Gradient Boosting (XGBoost), Support Vector Machine (SVM), and Multilayer Perceptron (MLP). Through internal validation, the effectiveness of various model algorithms was compared, and their predictive capabilities were evaluated to determine the optimal model. Finally, an online calculation platform was developed based on the optimal model to facilitate the early diagnosis of patients with GIM.

## 2 Materials and methods

### 2.1 Patients population

Inclusion criteria were as follows: 1) Inpatients at the First Affiliated Hospital of Dalian Medical University from January to December 2023. 2) Patients who underwent gastroscopy and endoscopic biopsy during hospitalization. 3) Completed basic serological tests such as liver function, kidney function, and *H. pylori* testing during hospitalization. 4) Patients without serious diseases affecting the heart, lungs, liver, kidneys, or blood system. 5) Patients over 18 years of age who signed an informed consent form. Exclusion criteria were as follows: 1) Patients diagnosed with gastric cancer or other malignant tumors. 2) Patients with a history of gastric surgery. 3) Patients previously diagnosed with autoimmune gastritis and other autoimmune related disease.

**FIGURE 1:**
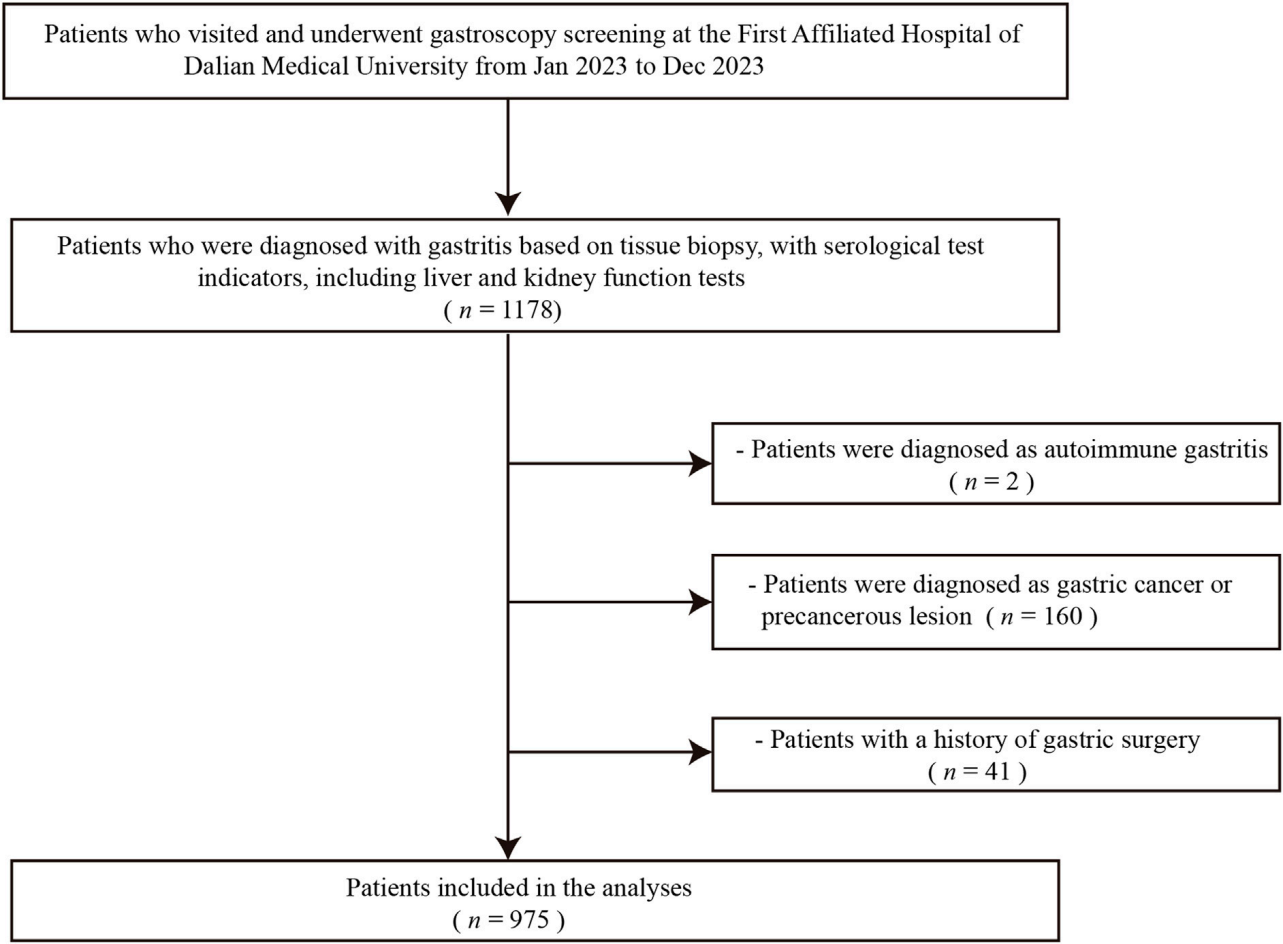
Flowchart depicting patients’ enrollment process. This flowchart illustrates the detailed screening and inclusion process of patients, highlighting the steps taken to ensure appropriate enrollment in the study. The process outlines the initial number of candidates screened, the criteria for inclusion and exclusion, and the final count of patients enrolled (n = 975), providing insights into the patient selection methodology used in the study.

As illustrated in Figure 1, this flowchart provides a detailed overview of the patient screening and inclusion process, facilitating an understanding of the methodology behind participant selection in this study. A total of 1,178 individuals meeting the criteria were screened for inclusion in this study cohort. Based on the exclusion criteria, 160 patients were diagnosed with gastric cancer or other malignant tumors, 2 patients were diagnosed with autoimmune-related gastritis, and 41 patients had undergone gastric surgical treatment. Therefore, a total of 975 patients met the criteria for inclusion in this study. The studies involving humans were approved by the institutional Ethics Review Board of First Affiliated Hospital of Dalian Medical University. The ethical approval number for this study is PJ-KS-KY-2024-574. The study were conducted in accordance with the Declaration of Helsinki.

### 2.2 Data collections

This study retrospectively reviewed electronic medical records and laboratory management systems to collect patient demographics, established potential predictors of gastric-related diseases, and common blood test indicators. The list of screened and enrolled patients was collected using the Yidu Cloud software of the First Affiliated Hospital of Dalian Medical University. Patient demographics included age, sex, BMI, family history of cancer, smoking history, and alcohol consumption habits. Established potential predictors of gastric-related diseases included *H. pylori* infection status, grading of gastric mucosal atrophy, gastric mucosal histopathology biopsy results, and gastroscopic findings such as bile reflux diagnosed by gastroscopy. The classification of gastric mucosal atrophy was based on the Kimura-Takemoto Classification (Kotelevets et al., 2021), with levels assigned as C1-C2 for grade 1, C3-O1 for grade 2, and O2-3 for grade 3. Additionally, grade 0 indicates the absence of gastric mucosal atrophy. In the gastric mucosal tissue samples, HE staining was used to observe whether the gastric mucosal epithelium contained cells similar to those of the small intestinal epithelium, such as columnar epithelium, goblet cells, or Paneth cells. In addition, if immunohistochemical staining was positive for small intestinal mucin (MUC2), intestinal metaplasia was diagnosed. The above data collection was performed by two independent researchers. Disagreements were resolved by a third researcher. Routine laboratory indicators included glucose (Glu), total bilirubin (TBIL) indirect bilirubin (IBIL), direct bilirubin (DBIL), total protein (TP), albumin (ALB), albumin/globulin ratio (ALB/GLO), prealbumin (PA), alanine aminotransferase (ALT), aspartate aminotransferase (AST), gamma-glutamyltransferase (GGT), cholinesterase (ChE), total bile acids (TBA), alkaline phosphatase (ALP), glycocholic acid (GCA), homocysteine (Hcy), estimated glomerular filtration rate (eGFR), creatinine (Crea), uric acid (UA), cystatin (Cys). Data collection for this article was retrospective, and missing data was inevitable. To avoid the impact of missing data on the analysis, we imputed the missing values. We first calculated the proportion of missing values for each variable. All variables had missing data rates below 10% (missingness 0%–2.8%). Then we imputed missing categorical data by the cohort mode and missing continuous data by the cohort median. In addition, among all evaluation indicators, those with a missing data rate of 10% or higher were excluded from the analysis. Finally, the data was standardized. Data extraction and cleaning were performed using R software.

### 2.3 Predictive model construction and evaluation

The patients were randomly divided into a training dataset and a testing dataset in a 7:3 ratio. Before modeling, variable selection was conducted on training set. Then, we employed a comprehensive suite of eight machine learning algorithms to develop robust predictive models. These algorithms were carefully selected to encompass a diverse range of approaches, from traditional statistical methods to advanced ensemble techniques and neural networks. The implemented models include: Decision Tree (DT), Elastic Net (ENet), K-Nearest Neighbors (KNN), LightGBM, Random Forest (RF), eXtreme Gradient Boosting (XGBoost), Support Vector Machine (SVM), and Multilayer Perceptron (MLP). Model training employed five-fold cross-validation on the training set and Hyperparameters for each model are provided in Supplementary Table S1. The predictive performance of each model was evaluated using ROC curves in both the training and testing datasets (Obuchowski and Bullen, 2018; Cabot and Ross, 2023; Van Calster et al., 2018). The prediction model with the best performance on the testing set was ultimately selected (Vickers et al., 2016). The interpretation of the prediction model was carried out using SHAP (Shapley Additive exPlanations) method, which accurately calculates the contribution and impact of each feature on the final prediction (Li et al., 2023). We computed SHAP with the R package fastshap. For global importance, we summarized feature importance as mean(|SHAP|) and displayed it with horizontal bar plots. In summary plots, for continuous features, we used beeswarm-style summary plots to show the distribution of SHAP values for each feature. For categorical features, we visualized per-level SHAP distributions using boxplots with overlaid points. The SHAP values provide critical insights into the impact of individual features on model outcomes.

### 2.4 Statistical analysis

All statistical analyses and calculations were conducted using R version 4.2.2. Categorical variables are presented as totals and percentages, with group differences assessed using the chi-square test. Continuous variables following a normal distribution are expressed as means and standard deviations, whereas those not following a normal distribution are described using medians and quartiles. A t-test was employed for normally distributed variables, while the Mann-Whitney U test was used for non-normally distributed variables to compare these continuous variables between two groups. For all analyses, we considered a *p*-value of less than 0.05 to be statistically significant.

## 3 Results

### 3.1 The characteristics of patients

The total number of patients undergoing gastroscopy at First affiliated hospital of Dalian Medical University was 3,518 in 2023. Among them, 1,178 patients met the inclusion criteria. Based on the exclusion criteria, a total of 975 patients were included in the study. The specific flowchart is shown in Figure 1. Of these patients, 322 (32.98%) were pathologically confirmed to have GIM, while 653 (66.67%) belonged to the non-atrophic intestinal metaplasia group. The distribution of gastric mucosal atrophy severity in this study cohort, as classified by the Kimura-Takemoto system, exhibited a diverse pattern. The largest group comprised patients with grade 1 gastric mucosal atrophy (C1-C2), accounting for 50.57% of the population, representing mild atrophic changes. This was followed by patients with non-atrophic gastritis at 30.52%, indicating inflammation without significant atrophy. Grade 2 gastric mucosal atrophy patients constituted the third largest group at 15.93%, signifying moderate atrophic progression. Notably, grade 3 atrophy patients represented the smallest fraction at 2.98%, reflecting advanced atrophic changes. This distribution highlights a predominance of mild to moderate gastric mucosal alterations in the study population, with a substantial proportion showing early-stage atrophy or non-atrophic gastritis. This distribution pattern provides valuable insights for clinical practice.

The positive rate for *H. pylori* infection was 44.44%, with 28.40% of *H. pylori* eradication. The average age of the patients was 66 years, with 49.03% being male. The median BMI was 24.24. Among the patients, 16.31% had a smoking history, and 13.03% had a history of alcohol consumption. The incidence of cholecystitis and bile reflux was 4.92% and 6.15%, respectively, both under 10%. Serological test indicators, which did not follow a normal distribution, are presented using medians and quartiles. Table 1 summarizes patient characteristics and shows significant differences in gastric atrophy grading, *H. pylori* infection, gender, age, smoking history, alcohol history, crea, and GGT between GIM and non-GIM groups (*p* < 0.05). This suggests that these independent variables may be associated with GIM.

**TABLE 1:** The baseline characteristics of the study cohort.

Classification	Variable	Level	Overall (N = 975)	Gastric Intestinal Metaplasia (N = 322)	non-Gastric Intestinal Metaplasia (N = 653)	*p*
Demographics	Age		66.000 [59.000, 71.000]	67.000 [60.000, 72.750]	66.000 [59.000, 71.000]	0.0231
	Gender	Female	497 (50.97)	123 (38.20)	374 (57.27)	<0.0001
		Male	478 (49.03)	199 (61.80)	279 (42.73)	
	Body Mass Index (BMI)		24.240 [22.000, 26.560]	24.260 [22.225, 26.837]	24.240 [22.000, 26.470]	0.2808
	Smoking History	Neversmoked	803 (82.36)	244 (75.78)	559 (85.60)	0.0007
		Smoker	159 (16.31)	73 (22.67)	86 (13.17)	
		Formersmoker	13 (1.33)	5 (1.55)	8 (1.23)	
	Alcohol Consumption History	No	848 (86.97)	268 (83.23)	580 (88.82)	0.0194
		Yes	127 (13.03)	54 (16.77)	73 (11.18)	
Past Medical History	Biliary Tract Diseases (Cholelithiasis and Cholecystitis)	No	927 (95.08)	310 (96.27)	617 (94.49)	0.2914
		Yes	48 (4.92)	12 (3.73)	36 (5.51)	
	Helicobacter pylori Infection Rate	Negative	264 (27.16)	7 (2.17)	257 (39.54)	<0.0001
		Positive	432 (44.44)	203 (63.04)	229 (35.23)	
		Eradication	276 (28.40)	112 (34.78)	164 (25.23)	
Endoscopic Indicators	Mucosal Atrophy	No	297 (30.52)	2 (0.62)	295 (45.31)	<0.0001
		C1 - C2	492 (50.57)	180 (55.90)	312 (47.93)	
		C2 - O1	155 (15.93)	113 (35.09)	42 (6.45)	
		O2 - O3	29 (2.98)	27 (8.39)	2 (0.31)	
	Bile Reflux	No	915 (93.85)	306 (95.03)	609 (93.26)	0.3475
		Yes	60 (6.15)	16 (4.97)	44 (6.74)	
Laboratory Tests	Glucose (Glu)		5.120 [4.678, 5.990]	5.265 [4.738, 6.055]	5.090 [4.660, 5.935]	0.1044
	Total Protein (TP)		65.800 [62.500, 69.800]	65.850 [62.225, 69.800]	65.800 [62.500, 69.800]	0.9901
	Total Bile Acids (TBA)		3.700 [2.300, 5.900]	3.500 [2.300, 5.900]	3.700 [2.300, 5.900]	0.7986
	Alkaline Phosphatase (ALP)		70.000 [57.000, 85.000]	68.000 [56.000, 85.000]	70.000 [58.000, 84.750]	0.3309
	Indirect Bilirubin (IBIL)		4.500 [2.950, 8.600]	4.600 [3.000, 8.200]	4.400 [2.900, 8.600]	0.8402
	Total Bilirubin (TBIL)		12.000 [9.100, 15.600]	11.600 [9.000, 15.400]	12.200 [9.100, 15.800]	0.1937
	Direct Bilirubin (DBIL)		3.700 [2.400, 5.600]	3.700 [2.500, 5.600]	3.700 [2.400, 5.600]	0.9825
	Alanine Aminotransferase (ALT)		16.000 [12.000, 22.500]	17.000 [12.000, 24.000]	16.000 [12.000, 22.000]	0.175
	Aspartate Aminotransferase (AST)		18.000 [15.000, 22.000]	19.000 [16.000, 23.000]	18.000 [15.000, 22.000]	0.3346
	Albumin/Globulin Ratio (ALB/GLO)		1.600 [1.400, 1.800]	1.600 [1.400, 1.800]	1.600 [1.400, 1.800]	0.6415
	Albumin (ALB)		40.600 [38.300, 43.100]	40.500 [38.100, 43.200]	40.600 [38.500, 43.100]	0.754
	Gamma-Glutamyl Transferase (GGT)		20.000 [15.000, 30.000]	21.000 [15.000, 32.000]	19.000 [14.000, 30.000]	0.0316
	Homocysteine (HCY)		11.150 [9.300, 13.800]	11.300 [9.400, 14.765]	11.050 [9.203, 13.275]	0.1106
	Proalbumin (PA)		241.000 [212.000, 276.000]	244.000 [211.000, 279.000]	240.000 [212.000, 274.250]	0.6016
	Uric Acid (UA)		315.000 [258.750, 371.000]	316.000 [258.000, 372.000]	313.000 [259.000, 370.000]	0.7881
	Creatinine (Crea)		64.500 [54.000, 77.000]	68.000 [57.000, 79.000]	63.000 [53.000, 75.000]	0.0008
	Estimated Glomerular Filtration Rate (eGFR)		90.000 [86.140, 92.125]	90.000 [87.740, 91.457]	90.000 [84.510, 92.740]	0.9354
	Cereuloplasmin (CG)		1.360 [1.100, 1.600]	1.300 [1.100, 1.600]	1.385 [1.110, 1.600]	0.3962
	Cholinesterase (ChE)		376.000 [300.000, 7117.000]	373.000 [301.000, 6990.000]	376.500 [300.000, 7181.000]	0.6875

^1^Categorical data are expressed as percentages (%), while Continuous data are represented using the median and interquartile range (median [IQR]).

^2^Continuous data from the serological testing indicators, including Total Protein (TP) (g/dL), Total Bile Acids (TBA) (µmol/L), Alkaline Phosphatase (ALP) (U/L), Direct Bilirubin (DBIL) (mg/dL), Indirect Bilirubin (IBIL) (mg/dL), Alanine Aminotransferase (ALT) (U/L), Aspartate Aminotransferase (AST) (U/L), Albumin (ALB) (g/dL), Gamma-Glutamyl Transferase (GGT) (U/L), Homocysteine (HCY) (µmol/L), Proalbumin (PA) (mg/L), Uric Acid (UA) (mg/dL), Creatinine (CRE) (mg/dL), Estimated Glomerular Filtration Rate (eGFR) (mL/min/1.73 m²), Ceruloplasmin (CG) (mg/dL), and Cholinesterase (ChE) (U/L) are all based on standardized data.

^3^Chi-square tests were used for categorical variables to compare proportions.

^4^Continuous variables were analyzed using t-tests for normally distributed data and Mann-Whitney U tests for non-normally distributed data.

### 3.2 Independent clinical variable screening

**FIGURE 2:**
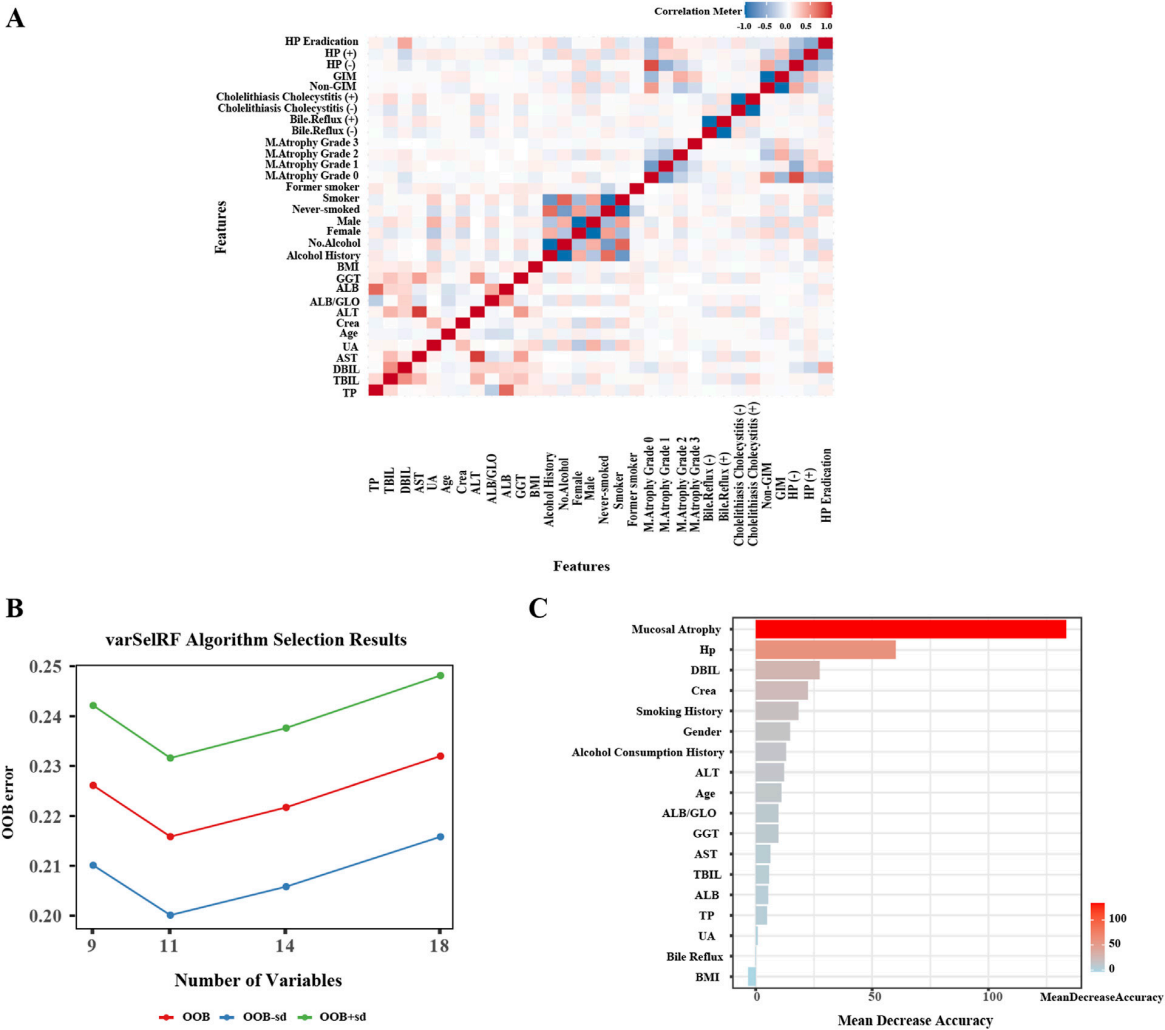
Independent variables Screening for gastric intestinal metaplasia. **(A)** Heatmap of correlation analysis between variables. **(B)** The VarSelRF algorithm calculates the OOB (Out Of Bag) standard error. **(C)** Evaluation of variable importance and rank them.

A correlation analysis of above mentioned variables is presented in Figure 2A, where a heatmap visualizes the strength of the relationships between variables, providing an initial indication of their intercorrelations. Subsequently, we employed the VarSelRF algorithm to assess the significance of these variables. The findings reveal that selecting 11 variables resulted in the lowest Out of Bag (OOB) error for the model (Figure 2B). These variables, ranked by importance (Figure 2C), are as follows: gastric mucosal atrophy, *H. pylori* infection, gender, DBIL, Crea, smoking history, alcohol history, ALT, age, ALB/GLO, and GGT. Based on these results, we have identified 11 critical clinical variables for predicting GIM, thereby providing robust evidence for constructing the predictive model.

### 3.3 Construction and evaluation of predictive models

**FIGURE 3:**
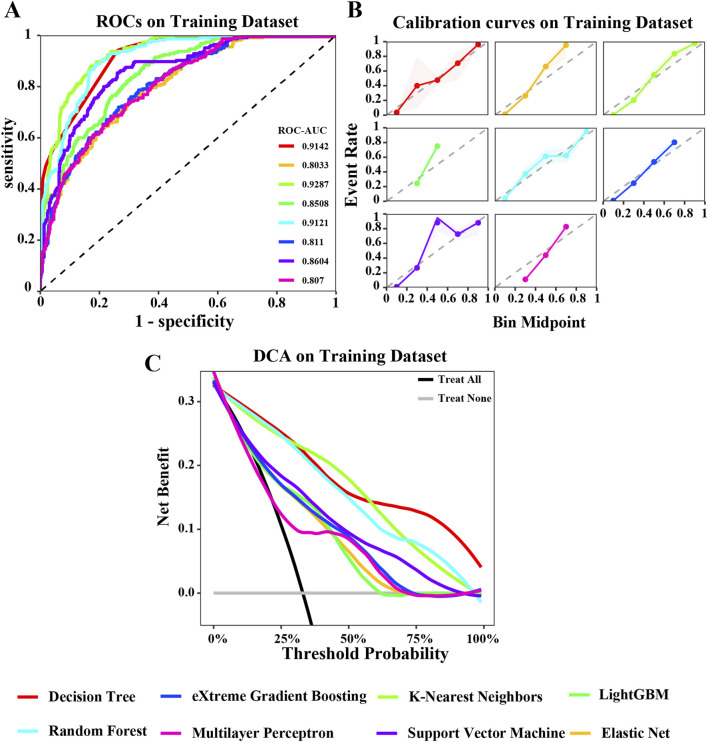
Evaluation of various machine learning algorithm models on the training dataset. **(A)** ROC curves illustrating the performance of each algorithm in the training dataset; **(B)** Calibration curves showing the predicted probabilities against actual outcomes in the training dataset; **(C)** Decision Curve Analysis (DCA) curves evaluating the clinical utility of the algorithms in the training dataset. The caption indicates that the different colored lines represent the following algorithms: Red for Decision Tree (DT), Blue for eXtreme Gradient Boosting (XGBoost), Light green for K-Nearest Neighbors (KNN), Green for LightGBM, Cyan for Random Forest (RF), Pink for Multilayer Perceptron (MLP), Purple for Support Vector Machine (SVM), Orange for Elastic Net (ENET).

**FIGURE 4:**
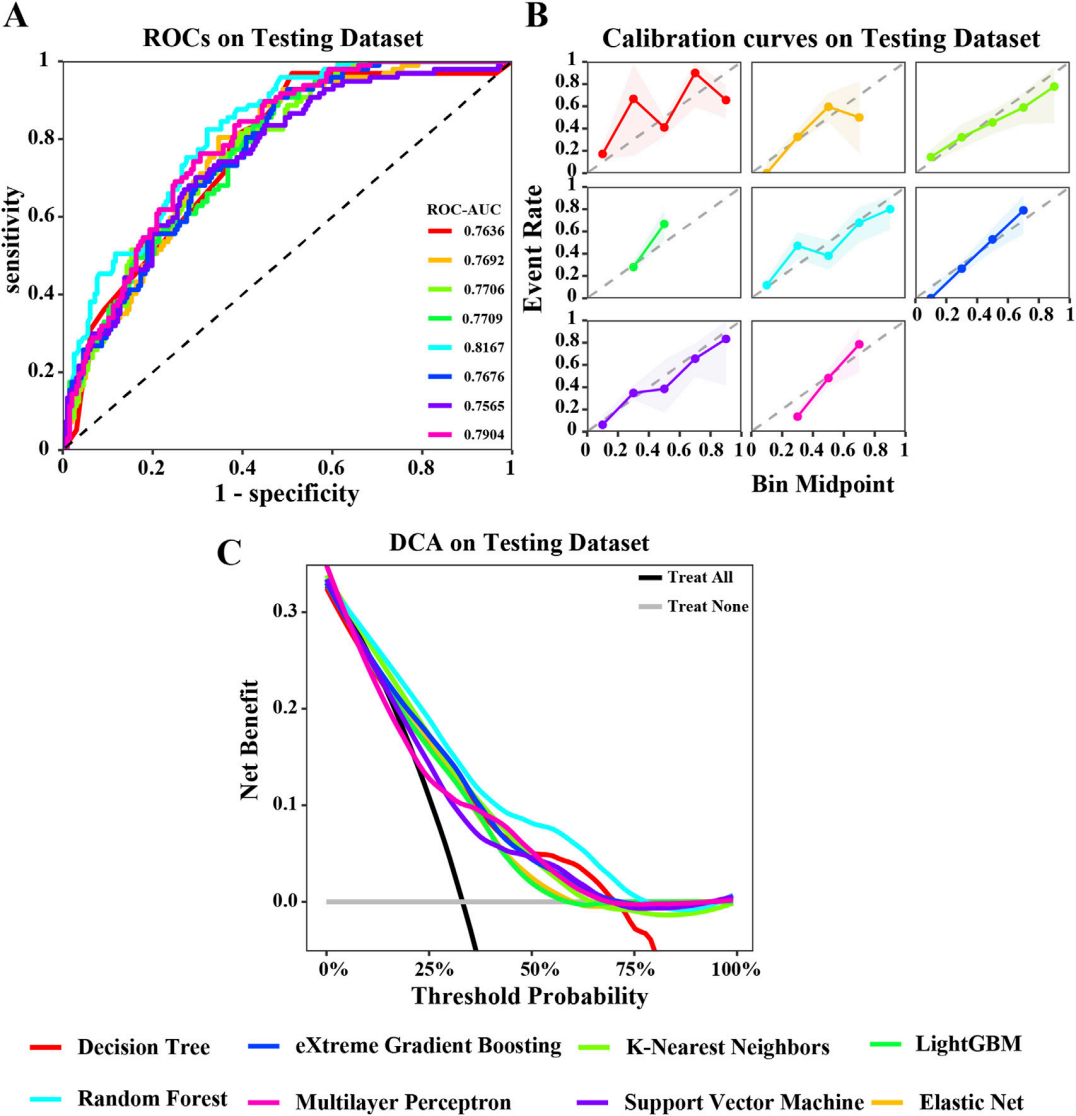
Evaluation of various machine learning algorithm models on the testing dataset. **(A)** ROC curves illustrating the performance of each algorithm in the testing dataset; **(B)** Calibration curves showing the predicted probabilities against actual outcomes in the testing dataset; **(C)** Decision Curve Analysis (DCA) curves evaluating the clinical utility of the algorithms in the testing dataset. The caption indicates that the different colored lines represent the following algorithms: Red for Decision Tree (DT), Blue for eXtreme Gradient Boosting (XGBoost), Light green for K-Nearest Neighbors (KNN), Green for LightGBM, Cyan for Random Forest (RF), Pink for Multilayer Perceptron (MLP), Purple for Support Vector Machine (SVM), Orange for Elastic Net (ENET).

To develop a prediction model for GIM, we utilized the 11 key variables identified previously. This study employed eight machine learning algorithms, including DT, ENet, KNN, LightGBM, RF, XGBoost, SVM, and MLP. The performance of models were comprehensively evaluated using various metrics such as ROC curve, calibration curve, and DCA curve. Figures 3, 4 illustrate the performance of the models on both the training and testing datasets.

In the training dataset, the ROC curve shows that most models perform well on the training dataset, fluctuating between 0.8033 and 0.9287. Among them, the areas under the ROC curves of KNN, DT, and RF models are all greater than 0.9 (Figure 3A). The calibration curve also suggests that, except for the significant deviation between the predicted probability and the actual probability of the SVM algorithm, most models have achieved good probability calibration on the training dataset. Especially the DT, ENet, KNN, RF, and XGBoost prediction models (Figure 3B). In addition, the DCA curve indicates that within most threshold ranges, most models such as DT, RF, KNN, SVM, etc. can benefit from extreme strategies such as “Treat all” or “Treat none” (Figure 3C). Subsequently, we tested these models in the testing dataset, and the area under the ROC curve of the RF model showed a maximum value of 0.8167 (Figure 4A). The calibration curve also indicated that the predictive model of the RF curve showed a good fit (Figure 4B), and the DCA curve showed that in most threshold ranges, the RF model achieved the highest net benefit in extreme strategies such as “Treat all” or “Treat none” (Figure 4C).

**FIGURE 5:**
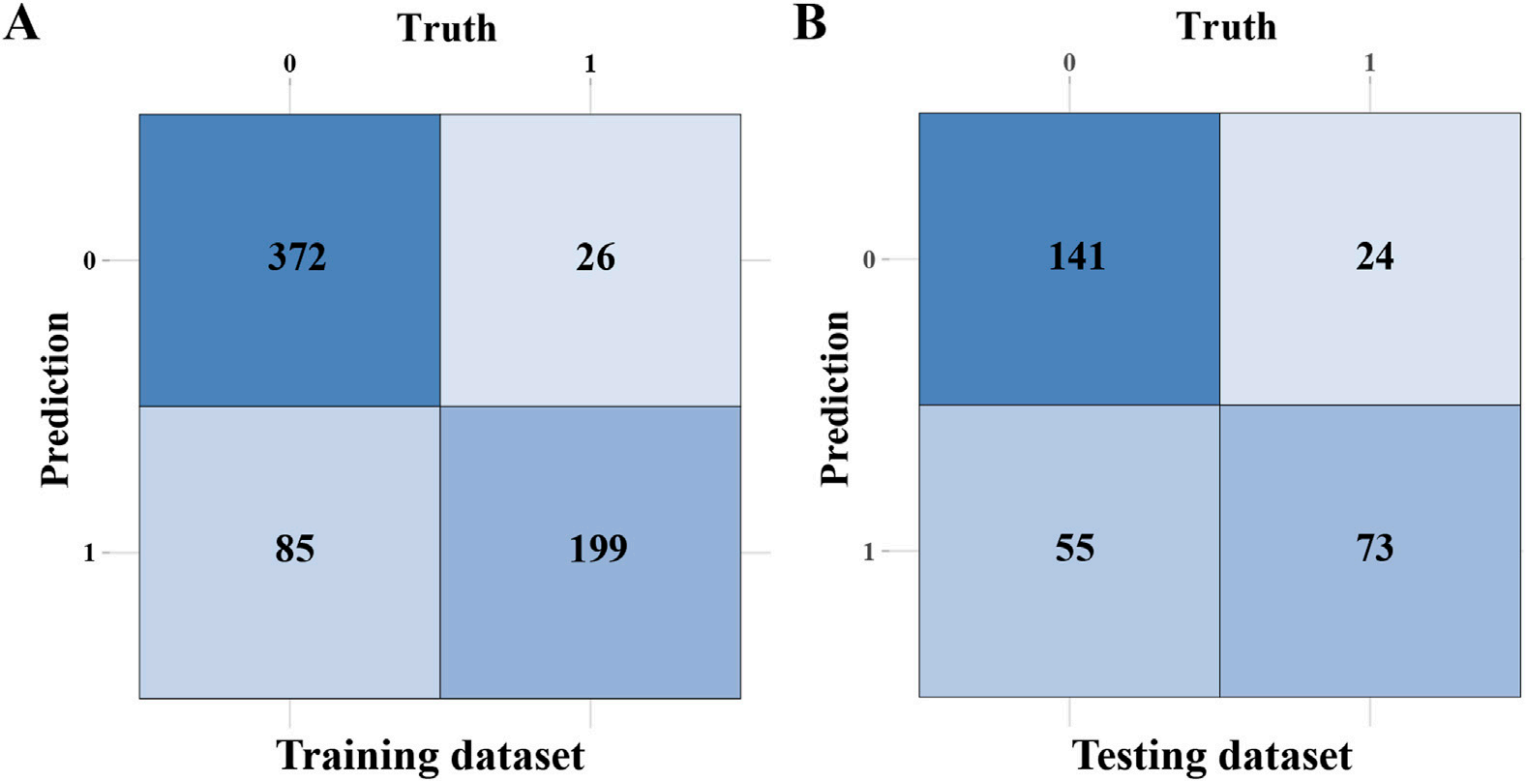
The confusion matrices of the RF model. **(A)** The confusion matrix of the RF model in training dataset. **(B)** The confusion matrix of the RF model in testing dataset.

To clearly show the prediction capability of the RF model, Figures 5A,B present the confusion matrices for the RF model in training and testing datasets, respectively. In the training set (n = 682), the random forest (RF) model correctly classified 372 non-intestinal metaplasia cases and 199 gastric intestinal metaplasia (GIM) cases. The model demonstrated robust performance on the training data, with a sensitivity of 70.1%, specificity of 93.5%, positive predictive value (PPV) of 88.4%, negative predictive value (NPV) of 81.4%, and an overall accuracy of 83.7%. In the testing set (n = 293), the model accurately predicted 141 of 165 non-GIM cases and 73 of 128 GIM cases. The testing results yielded a sensitivity of 57.0%, specificity of 85.5%, PPV of 75.3%, NPV of 71.9%, and an accuracy of 73.0%. These findings indicate robust performance of the RF model in predicting GIM. Through a comprehensive evaluation, we validated the effectiveness of the eight machine learning algorithms in constructing predictive models for GIM. We analyzed the performance and clinical applicability of each model from multiple perspectives. Based on model evaluations, particularly their performance on testing datasets, we found that the RF model excelled in prediction accuracy and stability. By aggregating multiple decision trees via bootstrap sampling (bagging) and averaging their predictions, RF achieves superior generalization performance. Consequently, we selected the RF algorithm as the predictive model for GIM in this study.

### 3.4 Interpretability analysis of the RF prediction model

In the application of machine learning models, elucidating the decision-making process and quantifying the contribution of individual features to predictive outcomes are crucial for clinical interpretability. SHAP values offer a theoretically consistent and clinically intuitive framework for model interpretation. This approach conceptualizes each feature as a “contributor” to the predictive outcome, employing cooperative game theory principles to fairly allocate the “prediction impact” among all features. Through SHAP-based analysis, clinicians can not only identify the relative importance of predictive variables but also discern their directional influence on model outputs, thereby enhancing the clinical utility and trustworthiness of machine learning applications in medical decision-making.

**FIGURE 6:**
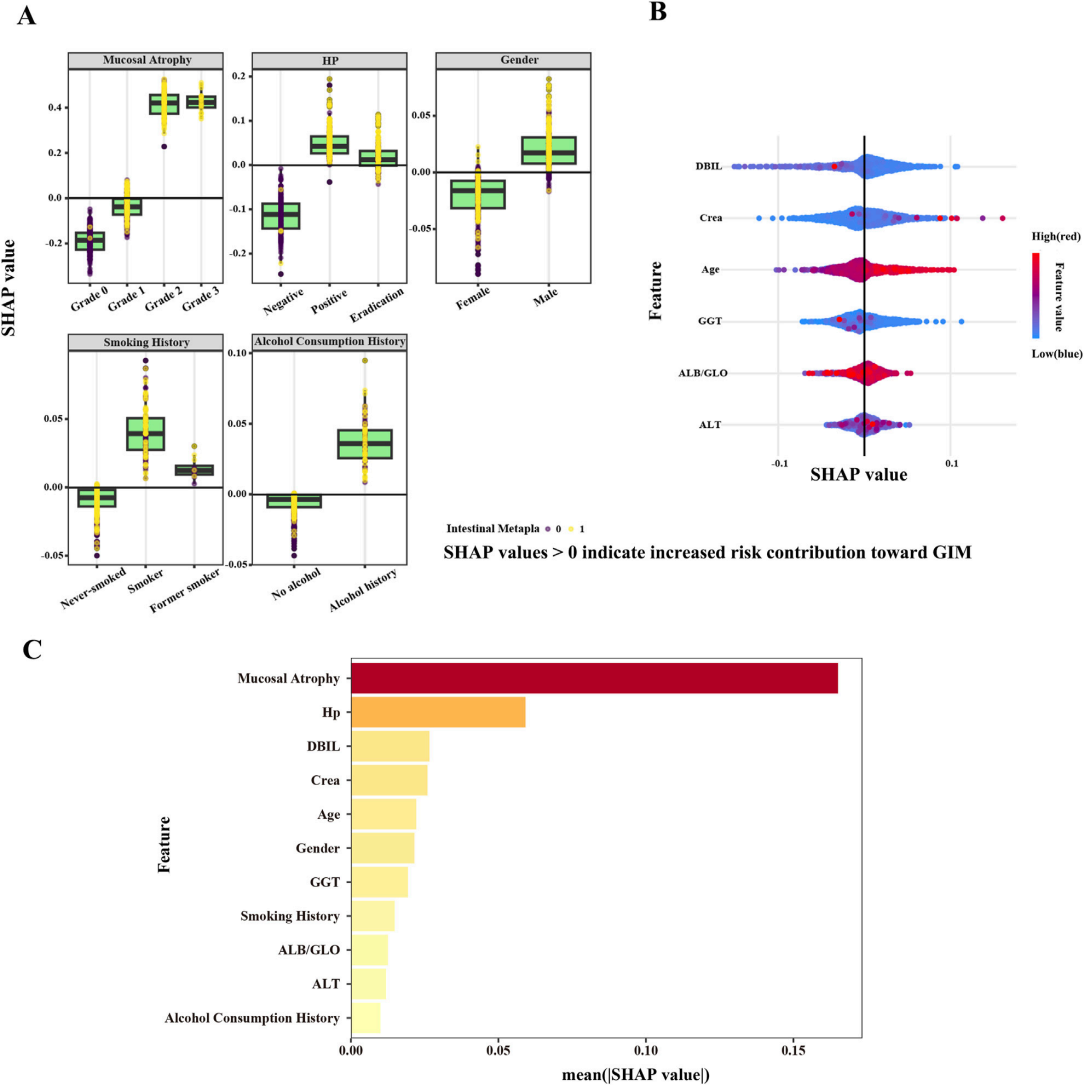
SHAP values based on RF model. **(A)** SHAP values of categorical variables. **(B)** SHAP values of continuous variables. SHAP values >0 indicate increased risk contribution toward GIM, while a SHAP value <0 indicates a risk factor that inhibits GIM outcomes. The color gradient represents the feature value, transitioning from blue (low feature value) to red (high feature value). **(C)** The mean SHAP value of all variables.

In this study, SHAP values were utilized to determine the roles of 11 independent variables in the RF model (Figure 6). Figure 6A illustrates the SHAP values of categorical variables, including the grade of gastric mucosal atrophy, *H. pylori* status, gender, smoking history, and alcohol history. A SHAP value greater than 0 for an independent variable suggests a promoting effect on GIM outcomes, whereas a value less than 0 indicates an inhibitory effect. Gastric mucosal atrophy at grade 2 or grade 3 are identified as risk factors for GIM, whereas grade 0 and grade 1 serve as protective factors. Factors such as *H. pylori* infection, successful *H. pylori* eradication, being male, smoking, being a former smoker, and having a history of alcohol consumption all positively contribute to the pathogenesis of GIM outcomes. In contrast, the absence of *H. pylori* infection, being female, and having no history of smoking or drinking indicate a negative impact on the pathogenesis of GIM outcomes. We also illustrate the SHAP values for continuous variables (Figure 6B), with red representing smaller observed values and blue indicating larger ones. In general, a higher observed SHAP value corresponds to a greater risk of GIM. Variables such as age, the ALB/GLO ratio and Crea are positively correlated with the occurrence of GIM, while DBIL and GGT are negatively correlated with the occurrence of GIM. As for ALT, it did not significantly demonstrate either a positive or negative effect on GIM outcomes regardless of whether the SHAP value was high or low.

### 3.5 Establishment of a web-based calculator

**FIGURE 7:**
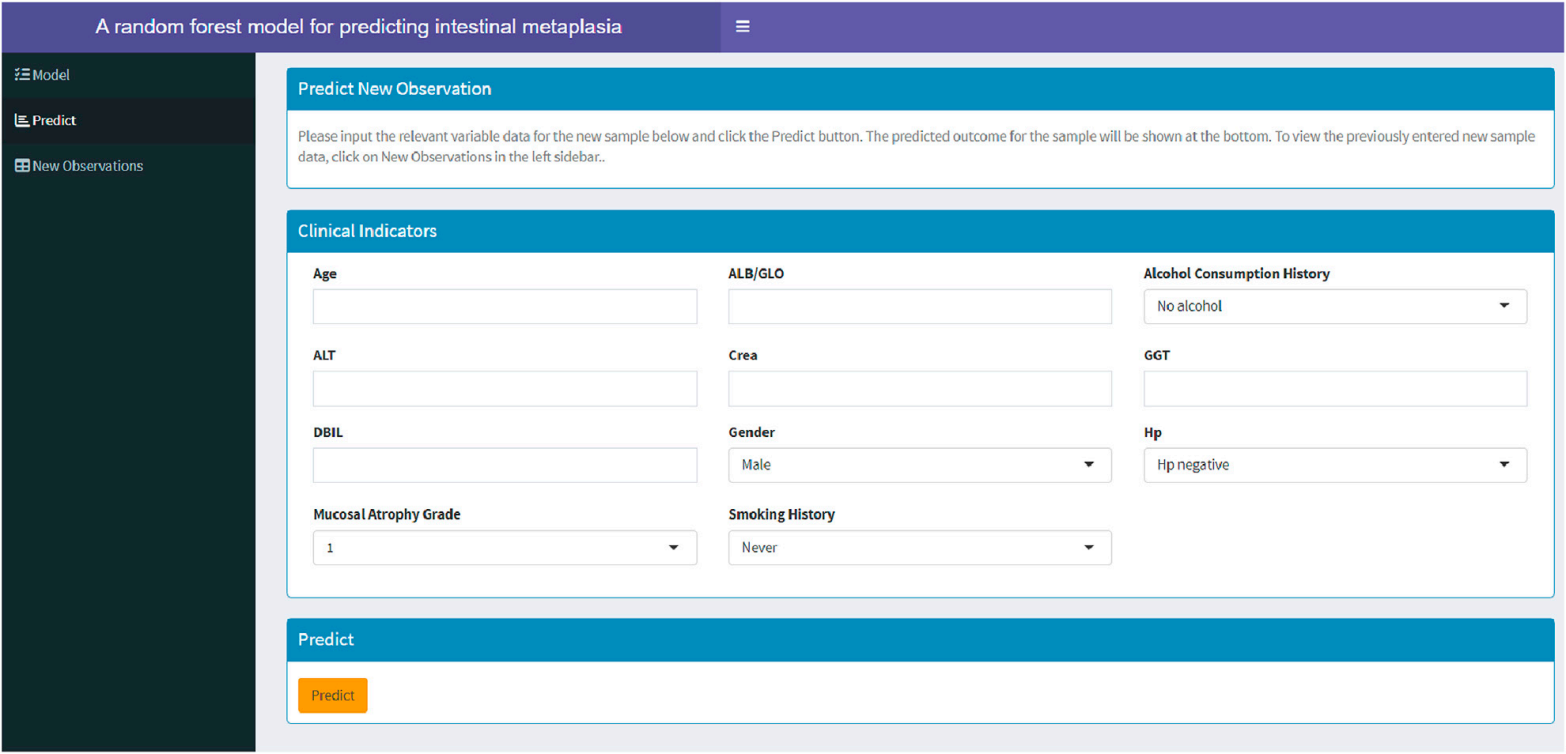
A web-based calculator for predicting GIM based on RF model.

Among the models constructed using eight machine learning algorithms, the RF model demonstrated superior performance. To assist clinicians in assessing the risk of GIM in patients and determining the necessity of gastric endoscopy biospy, this study developed a web-based calculator based on the RF model (https://fahdmu.shinyapps.io/GIMprediction/). This tool aims to enhance clinical decision-making by providing an efficient and accessible platform for GIM risk evaluation (Figure 7).

## 4 Discussion

Gastric intestinal metaplasia (GIM) represents a pivotal precancerous disease in the gastric carcinogenesis cascade, serving as a potential critical biomarker for early gastric cancer development. The timely identification of GIM enables effective surveillance, facilitates early intervention, and ultimately enhances patient prognosis and quality of life. While endoscopic screening awareness has improved, the implementation of risk-stratified screening strategies for high-risk GIM populations offers dual benefits: optimizing the diagnostic yield of endoscopic biopsies while simultaneously reducing healthcare expenditures and improving resource allocation efficiency.

To address the need for more precise risk stratification, this pioneering study developed a novel predictive model by integrating hepatorenal function biomarkers (GGT, DBIL, Crea, ALB/GLO, ALT) with established risk factors including gastric mucosal atrophy grading and *H. pylori* infection status. Through comprehensive evaluation of eight distinct machine learning algorithms, the RF model emerged as the optimal predictor, demonstrating superior performance metrics across both training and validation datasets compared to alternative approaches. This model not only enhances the identification of high-risk GIM individuals but also provides a foundation for personalized intervention strategies, thereby improving clinical outcomes and resource utilization.

The development of GIM is characterized by the progressive replacement of gastric mucosal cells with intestinal epithelial cells, a process that fundamentally alters the cellular microenvironment. This transformation not only disrupts normal tissue architecture but also creates conditions conducive to cellular dysplasia. The microenvironmental changes are further exacerbated by alterations in blood supply, which stimulate gastric mucosal epithelial cells through the release of inflammatory factors. Simultaneously, the repair capacity of these epithelial cells is critically dependent on their nutritional status, highlighting the intricate interplay between systemic factors and local tissue responses.

Given the systemic nature of these changes, molecular indicators in the blood emerge as valuable biomarkers for monitoring early GIM progression. Recognizing the clinical relevance of this approach, and considering the shared risk factors between gastric-related diseases and hepatorenal disorders, this study focused on clinically accessible serological indicators of liver and kidney function. The rationale for this selection is further supported by the fact that gastric mucosal repair and function are heavily reliant on the supply of nutrients through the bloodstream.

The interpretability of medical diagnostic models is critical for physician acceptance and clinical implementation. In this study, SHAP value analysis was employed to assess model interpretability, revealing that gastric mucosal atrophy and *H. pylori* infection are the primary predictors of GIM. These findings align with established research (Tong et al., 2024; Arai et al., 2022; Iwaya et al., 2023), further validating the model’s clinical relevance. The Kimura-Takemoto classification provides additional context: grade 2 and grade 3 gastric mucosal atrophy are identified as significant promoting factors for GIM, whereas grade 1 atrophy does not exhibit the same association. This suggests that GIM development likely requires a more extensive background of gastric mucosal atrophy, underscoring the importance of assessing the severity of atrophy in risk stratification. However, the model’s reliance on prior information regarding the extent of gastric mucosal atrophy introduces a limitation in its applicability. This prerequisite highlights the necessity of initial gastric endoscopic mucosal biopsy, particularly for patients without prior endoscopic screening. Such an approach not only ensures accurate risk assessment but also reinforces the critical role of baseline endoscopic evaluation in a comprehensive gastric cancer prevention strategy. In summary, while the model’s dependency on prior endoscopic data may restrict its immediate applicability, it emphasizes the importance of integrating endoscopic evaluation into routine clinical practice for effective risk assessment and prevention of gastric cancer.

In this study, both *H. pylori* infection and eradication were identified as positive factors for GIM outcomes. Previous *H. pylori* infection may promote GIM by releasing effector proteins (e.g., CagA and VacA) (Wang et al., 2014; Polk and Peek, 2010; Peek and Blaser, 2002), causing irreversible gastric mucosal damage. Although *H. pylori* eradication had a significantly lower SHAP value than infection, indicating a weaker promoting effect, it remains clinically important. However, it should be noted that the lack of analysis regarding the timing of eradication and treatment adherence may introduce bias in these findings, as these factors could significantly influence the outcomes. Additionally, demographic and lifestyle factors such as male gender, older age, smoking history, and alcohol consumption were significant contributors to GIM outcomes (Yuan et al., 2023; Liu et al., 2024; Tan et al., 2021). High ALB/GLO ratios and abnormal levels of DBIL, GGT, and ALT were also identified as independent risk factors. Impaired liver function may reduce the synthesis of albumin and antioxidants (e.g., glutathione), weakening gastric mucosal repair capacity and exacerbating damage. Clinical studies have shown that gastritis patients are more prone to hypoalbuminemia and elevated fibrinogen levels (Aziz et al., 2022). The ALB/GLO ratio, it reflects the balance between synthetic function (albumin) and immune or inflammatory activity (globulins), representing the body’s nutritional status and immune capacity. Albumin functions as a major plasma antioxidant, and reduced levels intensify oxidative damage to the gastric epithelium (Zhang et al., 2020). This balanced ratio may play a crucial role in the gastric stem cell niche and significantly influences GIM development. As for GGT, it serving as a key enzyme in glutathione metabolism, exhibits increased expression that indicates heightened oxidative stress and contributes to gastric mucosal damage, a recognized driving factor in gastric carcinogenesis (Salvatori et al., 2023). Furthermore, abnormal bile acid metabolism, particularly in bile reflux (e.g., deoxycholic acid), impairs gastric mucosal repair by inhibiting the FXR receptor and downregulating tight junction protein and TFF1 expression (Zhou et al., 2018). Elevated creatinine levels reflect impaired renal clearance function, leading to the accumulation of pro-inflammatory cytokines (IL-1β, TNF-α, IL-6) that can systemically affect gastric mucosa and promote metaplastic changes (Li et al., 2024; Teng et al., 2023; Jones et al., 2015; Wang et al., 2022). These findings align with our results, highlighting the multifaceted mechanisms underlying GIM development.

The innovation of this study is highlighted in two key aspects. First, it incorporates objective serum markers into the GIM prediction process and establishes an interpretable machine learning model. This approach not only enhances the model’s transparency and user understanding but also sheds light on a potential mechanistic link between hepatorenal function and GIM. Second, the study introduces a simple, accurate, and continuous GIM prediction tool designed to assist primary care physicians in the initial screening of high-risk populations. Unlike existing GIM prediction models, which predominantly rely on the technical expertise required for chromoendoscopy or electronic chromoendoscopy, this model significantly reduces the dependency on advanced equipment and specialized endoscopic skills. This innovation has the potential to predict GIM occurrence in advance, guide clinicians in determining the optimal timing for gastric mucosal biopsies, facilitate timely interventions, and ultimately improve patient outcomes.

However, this study is not without limitations. First, the lack of multi-center clinical samples hindered external validation of the model, which is crucial for generalizing its applicability. Second, the retrospective collection of serological indicators inevitably resulted in missing data. Although missing data were supplemented, this approach may still constrain the exploration of risk factors and underlying mechanisms associated with GIM. Future directions should focus on conducting multi-center validation studies to evaluate the model’s applicability across different populations and clinical settings. Additionally, prospective cohort studies could play a vital role in systematically collecting clinical data on risk factors associated with GIM. Furthermore, integrating this predictive model with endoscopic findings has the potential to improve diagnostic accuracy, helping clinicians determine the optimal timing for gastric mucosal biopsies and facilitating timely interventions. These actions would greatly enhance the model’s refinement and maximize its utility in clinical practice.

## 5 Conclusion

In summary, we developed a RF model to predict GIM by integrating demographic information, medical history, and clinical findings. This study highlights the innovative application of serological indicators as significant predictors of GIM development, revealing a potential link between hepatorenal function and GIM. Importantly, this tool may enable early identification of at-risk patients who could benefit from surveillance endoscopy, addressing the limitations of invasive screening methods. Additionally, we created a web-based calculator to assist clinicians in efficiently identifying high-risk populations, enhancing clinical decision-making and improving patient outcomes.

## Data Availability

The original contributions presented in the study are included in the article/Supplementary Material, further inquiries can be directed to the corresponding author.
